# Pharmacokinetic Profile of Oral Administration of Mefloquine to Clinically Normal Cats: A Preliminary In-Vivo Study of a Potential Treatment for Feline Infectious Peritonitis (FIP)

**DOI:** 10.3390/ani10061000

**Published:** 2020-06-08

**Authors:** Jane Yu, Benjamin Kimble, Jacqueline M. Norris, Merran Govendir

**Affiliations:** Sydney School of Veterinary Science, Faculty of Science, The University of Sydney, Sydney, NSW 2006, Australia; benjamin.kimble@sydney.edu.au (B.K.); jacqui.norris@sydney.edu.au (J.M.N.); merran.govendir@sydney.edu.au (M.G.)

**Keywords:** mefloquine, feline infectious peritonitis, pharmacokinetic, coronavirus, calicivirus

## Abstract

**Simple Summary:**

In searching for antiviral agents against feline coronaviruses and feline caliciviruses, mefloquine, a human anti-malarial drug, has been demonstrated to reduce viral load of feline coronaviruses and feline calicivirus in infected cells. In this study, mefloquine was administered orally to seven clinically healthy cats twice weekly for four doses and mefloquine concentrations in blood were measured to investigate the pharmacokinetic profile—the movement of drug in the body. The maximum blood concentration of mefloquine was 2.71 ug/mL and was reached 15 h after a single oral dose was administered. Mefloquine side effects included vomiting following administration without food in some cats, and mild increases in symmetric dimethylarginine (SDMA), an early kidney biomarker. This study provides valuable information on mefloquine’s profile in cats as an introductory step towards investigating it as a potential treatment for feline coronavirus and feline calicivirus infection in cats.

**Abstract:**

The pharmacokinetic profile of mefloquine was investigated as a preliminary study towards a potential treatment for feline coronavirus infections (such as feline infectious peritonitis) or feline calicivirus infections. Mefloquine was administered at 62.5 mg orally to seven clinically healthy cats twice weekly for four doses and mefloquine plasma concentrations over 336 h were measured using high pressure liquid chromatography (HPLC). The peak plasma concentration (Cmax) after a single oral dose of mefloquine was 2.71 ug/mL and time to reach Cmax (Tmax) was 15 h. The elimination half-life was 224 h. The plasma concentration reached a higher level at 4.06 ug/mL when mefloquine was administered with food. Adverse effects of dosing included vomiting following administration without food in some cats. Mild increases in serum symmetric dimethylarginine (SDMA), but not creatinine, concentrations were observed. Mefloquine may provide a safe effective treatment for feline coronavirus and feline calicivirus infections in cats.

## 1. Introduction

Feline coronavirus (FCoV) is an alpha coronavirus which occurs in two distinct pathotypes that can be distinguished by their biological behaviour, but not by their morphology [1]. Although the two pathotypes belong to the same virus species, different names have been used—feline enteric coronavirus (FECV) and feline infectious peritonitis (FIP). FECV is highly contagious by the faecal oral route. The infection is mostly asymptomatic or can cause mild diarrhoea [1,2]. FIP is a fatal, immune-mediated disease induced by virulent biotypes of FCoV known as feline infectious peritonitis virus (FIPV) in domestic and wild cats. Once cats with FIP show clinical signs, the median life expectancy ranges from days to weeks for the effusive form, and weeks to months for the non-effusive form [3,4,5,6]. However, there are a small number of cats who may survive for several years [7,8]. Traditionally, treatment options have been limited; however, recent experimental treatments using protease inhibitors and nucleoside analogs have produced promising outcomes [9,10,11,12,13], although these treatment options are not yet registered for cats. The lack of available treatment options escalates the need to investigate additional affordable antiviral treatments that are currently accessible. Additionally, there is therapeutic value in finding treatments that target different aspects of FCoV replication, as combination therapy in other viral infections is associated with higher rates of pathogen suppression and minimisation of antiviral resistance development [14,15]. 

Feline calicivirus is an important and common cause of upper respiratory tract disease and oral ulceration in cats, with more virulent forms of the virus recently emerging resulting in outbreaks of a systemic disease that is commonly fatal and like FIP, lacks effective antiviral treatments. [16]. 

In searching for antiviral agents against feline coronavirus and feline calicivirus, mefloquine, a human anti-malarial drug, was shown to inhibit viral load of FCoV in infected Crandell feline kidney cells without cytotoxic effects [17]. Its inhibition of cytopathic effects and viral replication at low concentrations support further investigation of this drug as a potential antiviral therapeutic agent. Our previous project developed an in vitro model to determine the extent and rate of hepatic clearance (Cl) of mefloquine [18]. Mefloquine undergoes phase I hepatic metabolism but not phase II conjugative metabolism in the cat [18]. There is no evidence that mefloquine should have delayed elimination in clinically healthy cats. Mefloquine plasma protein binding is approximately 99% in both feline plasma from clinically normal cats and plasma from cats with FIP [19].

As mefloquine is currently used for prophylaxis for malaria, information on its disposition, including drug absorption, distribution and rate and extent of elimination, is available for human adults and infants, with some information in dogs [20], but there is no information on mefloquine’s pharmacokinetic (PK) profile in cats other than the plasma protein binding at this stage [19]. Therefore, to assess whether mefloquine administration has any therapeutic advantage as an antiviral agent, determining the pharmacokinetic profile of mefloquine in clinically normal cats is necessary so that a dosage and dose frequency can be developed. Knowing the pharmacokinetic profile of mefloquine in the clinically normal cat is the transitional step that bridges preclinical mefloquine observations to medicating cats with mefloquine in the future. The aim of this study was to investigate the pharmacokinetic profile of mefloquine when administered orally at 62.5 mg (10–12 mg/kg) twice weekly. The second objective was to document any changes in haematological and/or biochemical analytes and physiological responses to mefloquine at this dosage regimen.

## 2. Materials and Methods

### 2.1. Animals

Eight adult cats (4 females, 4 males) were recruited by Invetus Pty Ltd. (Casino, NSW, Australia), an animal research facility, from their clinically normal stock animals. Body weights ranged from 5.0 to 5.8 kg (average 5.4 kg). The cats were aged 3–7 years (average 5.5 years). The cats were selected based on clinically normal physical examination, normal body condition score and body weight ≥5 kg. Exclusion criteria included cats with abnormal physical examination findings, cats that were underweight or those that were receiving medication. Seven cats were initially selected to enter the study. The cats were housed individually in pens in a cattery and supplied with food and water ad libitum. The selection of the clinically normal cats, their dosing with mefloquine, blood collection and housing were performed by Invetus. This study was approved by Wongaburra Research Centre Animal Ethics Committee as project USY F 18120 W on the 29th August 2019 and by The University of Sydney Animal Ethics Committee as protocol 2019/1662.

### 2.2. Drug Administration and Sample Collection

To collect blood, cats were mask induced and anaesthetised with isoflurane in 100% oxygen and 2–4 mL of blood was obtained from the jugular veins using a 22-gauge needle. Blood was collected in lithium heparin tubes, ethylenediaminetetraacetic acid (EDTA) tubes and serum tubes for mefloquine concentration quantification, haematology and biochemistry, respectively. A 250 mg mefloquine tablet (Lariam, Roche, Millers Point, NSW, Australia) was quartered along the tablet’s scored lines. A quarter tablet, or 62.5 mg mefloquine, was the dose for each cat. The weight of each quarter tablet was recorded for dosing consistency. Mefloquine (62.5 mg) was administered to each cat orally on days 0, 4 (corresponding to 96 h), 7 (168 h) and 10 (240 h). The cats were then monitored for 2 h after dosing for any adverse reactions. To determine mefloquine plasma concentration, serial blood samples were collected into lithium heparin tubes at 0 (pre-treatment), 1, 2, 4, 8, 12, 24, 48, 96, 168, 240 and 336 h after drug administration. On mefloquine administration days other than the first administration (t = 96, 168 and 240 h), blood samples were collected prior to dosing, and mefloquine was then administered, followed by wet or dry food administered within minutes after mefloquine dosing. Blood was also collected into EDTA and serum tubes for haematology and biochemistry at 168 and 336 h. Samples were centrifuged within 1 h of blood collection. Plasma and serum were immediately stored in a freezer (−20 °C) within 90 min of blood sampling. EDTA tubes were immediately sent to Idexx laboratory East Brisbane, Qld. Australia, for haematological analysis. Serum tubes were sent to Veterinary Pathology Diagnostic Services, The University of Sydney and the Idexx reference laboratory for biochemistry analysis. Serum was also sent to the Idexx reference laboratory for serum symmetric dimethylarginine (SDMA) and creatinine. For the determination of mefloquine concentration in the plasma, samples were batched and all samples were analysed by the veterinary pharmacology laboratory, Sydney School of Veterinary Science, The University of Sydney, within two months of blood collection. 

### 2.3. Drug Analysis Method and Sample Processing

Samples were quantified for mefloquine concentration by high pressure liquid chromatography (HPLC) with plasma samples modified from a validated method [19]. 

#### 2.3.1. Chemicals

Mefloquine, verapamil (as the internal standard [IS]), sodium phosphate, trimethylamine, phosphoric acid were purchased from Sigma-Aldrich (Castle Hill, Sydney, NSW, Australia). HPLC-grade acetonitrile and methanol were purchased from Thermo Fisher Scientific (Macquarie Park, NSW, Australia).

#### 2.3.2. HPLC Conditions

The HPLC system consisted of a Shimadzu LC-20AT delivery unit, DGU-20A3 HT degassing solvent delivery unit, SIL-20A auto injector, SPD-20A UV detector and CTO-20A column oven. Shimadzu LC Solution software (Kyoto, Japan) was used for chromatographic control, data collection and data processing. Chromatographic separation was performed with a Polaris C18-A column (5 µm, 150 × 4.6 mm) with a 1.0 mm Optic-guard C 18 pre-column (Choice Analytical, Thornleigh, NSW, Australia), with the column oven temperature set at 35 °C. The isocratic mobile phase contained a mixture of 25 mM sodium phosphate with 0.5% triethylamine adjusted to pH 6.0 with phosphoric acid, acetonitrile and methanol (50:25:25) at a flow rate of 0.8 mL/min. For each sample, the injection volume was 10 µL and the total run time was 15 min. The diode array detector was set at a wavelength of 220 nm. 

For sample preparation, mefloquine plasma concentrations of 0.156, 0.313, 0.625, 1.25, 2.50, and 10.0 µg/mL were prepared by serial dilution. The IS solution was prepared in 100% acetonitrile at a final concentration of 10 ug/mL. Pre-treatment feline plasma samples were used for the preparation of the standard curve. 

Simple protein precipitation using 100 µL of acetonitrile containing 10 µg/mL of IS was added to 100 µL of feline plasma samples to extract proteins from plasma samples. The samples were then vortexed and centrifuged at 14,000× *g* for 10 min. Ten microlitres of supernatant was injected into the HPLC system for analysis. 

### 2.4. Pharmacokinetic Analysis 

The data were evaluated using non-compartment analysis as the elimination phase was only evident at two time-points, i.e., 48 and 96 h. The mean peak plasma concentration (C max) and time to reach C max (T max) of the first dose were determined by visual inspection of the individual cats’ plasma concentration vs time curve over 96 h. The difference in the natural log of the plasma concentrations at 24 and 96 h, i.e., the slope of the curve from 24 to 96 h gave k_e_. The elimination half-life was estimated by ln 2/k_e_. The area under the curve (AUC_0-t_) at 96 h was calculated to the last measurable concentration using linear trapezoidal method. The apparent volume of distribution was calculated as:

V/F = (Dose/AUC) × (1/k_e_), (1)

where F is the bioavailability of the oral route and cannot be determined as the intravascular (IV) administration of mefloquine to cats has not been undertaken. The apparent clearance was calculated as:

Cl/F = V × k_e_. (2)

Area under the moment curve (AUMC_0–96h_) was calculated as the sum of the AUC when each of the concentration data points was multiplied by time. The mean residence time was calculated as 1/k_e._

### 2.5. Statistical Analysis

Two cats were excluded from statistical analysis due to vomiting. The mefloquine plasma concentration data points for the five cats (including Cat E) at 24, 96, 168, 240 and 336 h underwent Shapiro–Wilk normality test and all distributions were normal. However, SDMA concentrations were not normal at t = 0 h, but were normal at 168 and 336 h. Creatinine concentrations were normal at 0, 168 and 336 h. The mefloquine plasma mean concentrations were compared at 24, 96, 168, 240 and 336 h and underwent a repeated measures on-way ANOVA as did the mean SDMA and creatinine concentrations at 0, 168 and 336 h. Tukey’s multiple comparisons test was used to demonstrate whether the means at each time point were significantly different. Statistical analysis was accepted if *p* < 0.05. Statistical analysis was undertaken by Graphpad Prism 8 (San Diego, California, CA, USA). 

## 3. Results

**Figure 1:**
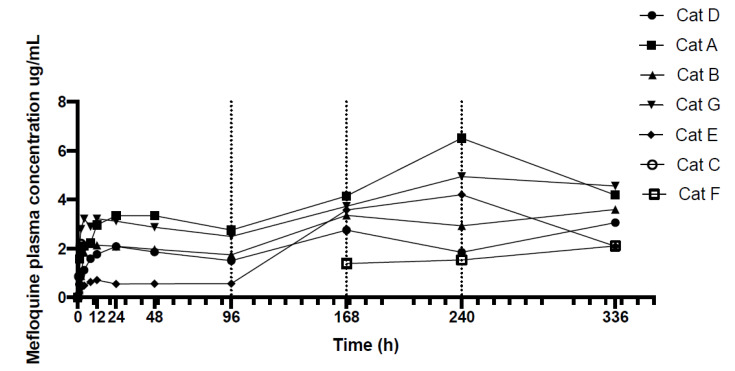
Mefloquine plasma concentrations (ug/mL) of seven cats over 336 h (14 days after initial dose). Cats were medicated with 62.5 mg per cat (10–12 mg/kg) after t = 0, 96, 168 and 240 h. Blood was taken just prior to treatment as denoted by vertical dotted lines.

**Figure 2:**
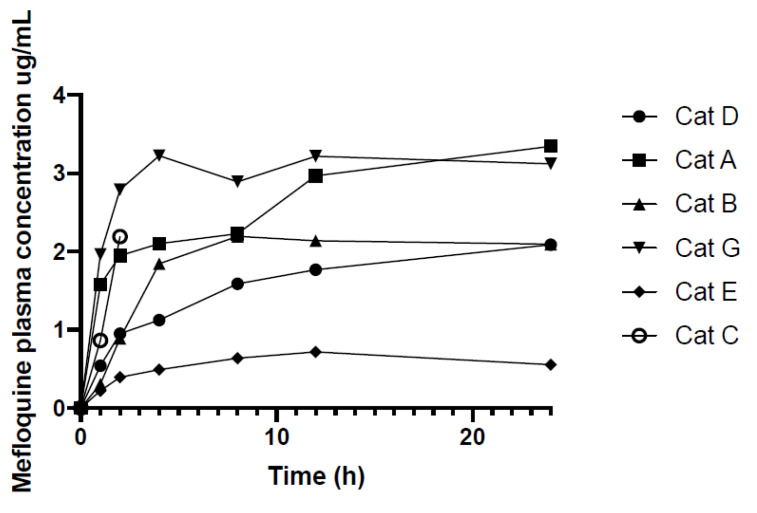
Mefloquine plasma concentrations (ug/mL) over the first 24 h after cats were administered 62.5 mg (10–12 mg/kg) mefloquine at t = 0.

Following administration of 62.5 mg of mefloquine per cat, the mean dose per kg was 11.8 mg/kg (median 12.3, range 10.8–12.5). The change in mefloquine plasma concentrations over 336 h (14 days) for seven cats are displayed in Figure 1 and the actual mefloquine plasma concentrations of each cat at each time point are provided in Table 1. Figure 2 shows the mefloquine plasma concentrations (ug/mL) over first 24 h. A single oral dose of mefloquine results in a Cmax of 2.71 µg/mL after the first dose, on average at 15 h (Tmax). Increases in mefloquine plasma concentrations were observed at 168, 240 and 336 h (Figure 1), after second dose administered just after 96 h, third dose administered just after 168 h and fourth dose administered just after 240 h, respectively, when mefloquine was administered with food, with the peak plasma concentrations reaching 4.06 µg/mL (mean) at 240 h. One cat (Cat C) vomited 15 min after dosing on day 0 (treatment 1). This cat was re-dosed with mefloquine on day 4 (treatment 2) but vomited approximately an hour after dosing, and was, therefore, removed from the study. Another cat (Cat F) vomited 5 min after dosing on day 1 (treatment 1). This cat was successfully dosed with mefloquine after feeding on the following treatment day (96 h) and was re-introduced into the study. No vomiting was observed after dosing this time. Blood samples were collected at 168, 240 and 336 h for Cat F, as shown in Table 1. 

The pharmacokinetic (PK) indices are provided in Table 2. Cats C and F were excluded from the PK analysis due to incomplete data. As Cat E’s PK profile skewed the data, indices for four cats (Cat A, B, D and G) with more consistent profiles were used for analysis. 

Haematology and serum biochemistry of six cats (Cat A, B, D, E, F and G) were performed prior to treatment (0 h), at 168 and 336 h. Cat C was removed from the study after the first two timepoints, and therefore blood collection was not continued. Haematology results were unremarkable in all six cats at all time points. Biochemistry results are illustrated in Table 3. 

**Figure 3:**
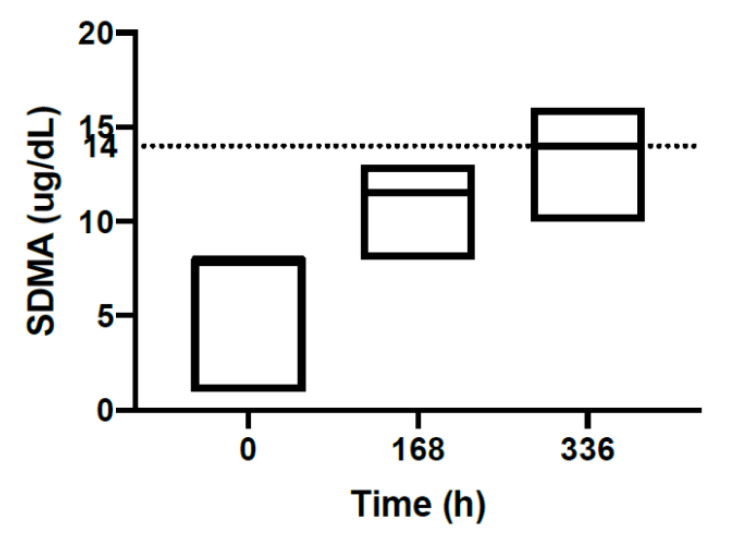
SDMA results (ug/dL) of six cats at 0, 168 and 336 h. The median SDMA concentration of each time point is shown with the upper and lower range. The dotted line represents the reference interval (0.00–14.0).

Biochemistry showed an increase trend in SDMA concentrations at 168 and 336 h in all six cats. A repeated measure one- way ANOVA, comparing SDMA at all time-points, was statistically significant *p* < 0.002. Tukey’s multiple comparisons tests show that mean SDMA at each time point was statistically different: 0 vs 168 h *p* = 0.002; 168 vs 336 h *p* = 0.005. Figure 3 shows the median SDMA concentrations at each time point with upper and lower range from six cats. The median SDMA at 0 h was 8.0 g/dL (range 1.0–8.0), median SDMA at 168 h was 11.5 g/dL (range 8.0–13.0) and that at 336 h was 14.0 g/dL (range 10.0–16.0). Creatinine concentrations did not differ significantly at 0, 168 and 336 h in six cats. Three cats had elevations in liver parameters including ALT, AST and ALP pre-treatment. Among these three cats, one cat (Cat G) had an ALT value as high as 161 U/L and ALP was 75.0 U/L pre-treatment, ALT and ALP remained elevated at 166 U/L and 87.0 U/L, respectively at 336 h. This cat had no adverse effects during the study. One cat with mildly increased ALT (142 U/L) was removed from the study due to vomiting (Cat C). Another cat with mildly increased ALT (144 U/L) and AST (83.0 U/L) at pre-treatment remained clinically healthy throughout the study and both liver parameters returned to normal range at 168 and 336 h. Other biochemical analytical changes were unremarkable. Blood glucose at 0 h was not provided, as artifactual hypoglycaemia was seen in all samples. This was suspected to result from a problematic collection method of blood glucose samples. 

Although Cat C and F vomited on the first administration of mefloquine, all other cats tolerated the medication well on subsequent administration when mefloquine was administered with food. No other adverse effects were observed in any cat over the two weeks of treatment. 

## 4. Discussion

This is the first pharmacokinetic study of mefloquine in cats. Mefloquine’s only reported use in animals is as an anti-malarial drug for raptors and penguins [21,22]. In addition to its use as an anti- malarial agent, successful treatment with this drug has been reported in people with progressive multifocal leukoencephalopathy caused by John Cunningham virus (JCV) [23,24]. Its antiviral action has been demonstrated in vitro in FCoV [17], feline calicivirus [25], dengue virus type 2 and Zika virus in people [26], and recently, pangolin coronavirus GX_P2X, a model for SARS-CoV-2, the cause of COVID-19 in people [27]. Its exact mechanisms of actions as an anti-malarial or antiviral agent are unknown [17,28,29]. 

As mefloquine is an anti-malarial prophylactic and treatment for people, its pharmacokinetic profile has been documented. In healthy human volunteers, mefloquine’s oral absorption half- life is 1–4 h (mean 2.1 h) [30]. The oral bioavailability of mefloquine in cats is unknown, as the required IV AUC required for the calculation has not been undertaken in cats. However, the oral bioavailability of mefloquine was found to be about 67–90% (mean 78%) in dogs [20]. Mefloquine reaches peak plasma concentrations after around 6–24 h (median 17.6 h) in people [30]. When administered orally to cats, the time to peak plasma concentrations (Tmax) is comparable to humans, on average 15 h. The estimated total apparent volume of distribution in healthy people is approximately 19.2–22.1 L/kg and systemic clearance is 0.026–0.042 L/h/kg [31], while the apparent mean ± SD volume of distribution in cats is 17.4 ± 4.08 L/kg, and apparent clearance is 0.060 ± 0.020 L/h/kg when calculated over 0 to 96 h. Plasma protein binding was 98% in healthy human volunteers and patients with uncomplicated falciparum malaria [30] and also 99% in feline plasma from clinically normal cats and plasma from cats with FIP [19]. In humans, the elimination half-life of mefloquine is approximately 20 days in healthy subjects, 10 to 14 days in patients with uncomplicated falciparum malaria [32,33] and 20 days in cases involving severe malaria [33,34]. In humans, it is recommended that there is a loading dose and then once weekly medication [35,36]. Mefloquine is excreted slowly from the body through faeces and urine [31,37]. Our study estimated that the elimination half-life of mefloquine in clinically healthy cats is approximately 224 h or approximately 9.3 days, similar to that of healthy people. The half-life calculation was only based on the 24 to 96 h timepoints; further studies sampling a single dose administration of mefloquine for longer than 96 h in cats may give a more definite result.

When mefloquine was administered with food, oral absorption was enhanced. The pharmacokinetic analysis showed that the mean plasma concentration was higher (4.06 ug/mL) at 240 h when mefloquine was given with food compared to plasma concentration of 2.71 ug/mL at 15 h when mefloquine was given without feeding. Other considerations for the higher plasma concentration at 240 h include the cumulative effect of the drug with multiple dosing and possible enterohepatic circulation of the drug. In humans, the presence of food in the gastrointestinal tract affects the pharmacokinetic properties of mefloquine by significantly increasing the rate and extent of absorption [38]. 

It has been demonstrated that mefloquine at a concentration of 10 µM showed marked inhibition towards two biotypes of FCoV, FIPV WSU 79-1146 (FIPV1146) and FECV WSU 79-1683 (FECV1683), acquired from the American Type Culture Collection (Virginia, USA), [17]. Given the molecular weight of mefloquine is 378 g/mol, a plasma concentration of 10 µM = 3.78 µg/mL is reached when 10 µM of mefloquine is used [17]. This study showed that when a single oral dose of mefloquine of ~12.5 mg/kg was administered, a peak plasma concentration (Cmax) of 2.71 µg/mL is reached. A higher dose of mefloquine may be required for the drug to inhibit the FIP virus. However, it is possible that when mefloquine is administered with food the Cmax will be much higher than that reported at 12 to 24 h. To assess the efficacy of mefloquine towards FCoV virus, a clinical trial is warranted. Additionally, mefloquine has been shown to inhibit cytopathic effects in cells infected by a SARS-CoV-2a-related pangolin coronavirus (GX_P2X), making it a potential drug for use in cats with SARS-CoV-2 infection [27].

Chloroquine, a 4-aminoquinoline with similar mode of action to mefloquine [39], has been demonstrated to have inhibitory effect against FIPV replication and anti-inflammatory effect in vitro and improved clinical score of experimentally induced FIP cats [40]. While mefloquine has been shown to inhibit FIPV in vitro [17], its clinical efficacy in cats with FIP remains unknown. Chloroquine has, however, caused an increase in ALT levels when used in FIP infected cats. In this study, mefloquine did not cause an increase in ALT levels. Although some cats had elevated ALT levels prior to mefloquine dosing, a further increase after four doses of mefloquine twice weekly was not seen. Hydroxychloroquine has been investigated in a clinical trial for COVID-19 treatment in people [41] and recently, its antiviral property against FIPV has also been studied in vitro [42]. When used with recombinant feline IFN-*ω,* hydroxychloroquine showed increased antiviral activity against FIPV infection [42]. Further clinical studies are needed to verify its clinical efficacy and safety in cats with coronavirus or calicivrus infections. The incidence of adverse effects following mefloquine administration in people is common with 47–90% of people experience some mild or moderate adverse effects [30,43,44]. The incidence of adverse effects decreases with prolonged use, from 44% during the first 4 months to 19% after one year [30,45]. The most common adverse effects include neuropsychiatric effects [46,47,48], gastrointestinal dysfunction [49], dermatological signs [50], haematological changes [51] or cardiovascular dysfunction [30,33]. In humans, nausea and vomiting are both common adverse effects and can be dose-dependent and age-related, with the highest risk in younger children [49,52]. In this study, two cats vomited following the first mefloquine administration without food. Mefloquine was successfully re-administered to one cat (Cat F) after feeding and this cat was therefore re-introduced into the study. Cat C was also re-dosed at the second mefloquine dosing (day 4); however, this cat refused food before the second dosing. Mefloquine was therefore dosed without food. This cat vomited again and was removed from the study. When mefloquine was administered with food, no further vomiting was observed. No other adverse effects were seen clinically in the cats in our study. However, our cats were only observed for 14 days. Any delayed or long-term adverse effects of mefloquine in cats remain unknown. It is also possible that the incidence of adverse effects might reduce with long term administration, as observed in people [30,45]. 

The cause of the lower plasma concentration curve of Cat E over the first dosing interval (0–96 h) was unknown (Figure 1 and Figure 2). An age difference may explain the lower plasma concentration curve of Cat D and E as these two cats are younger than the others (3 years old vs. 6–7 years old). Reduction in hepatic clearance, increase in volume of distribution of lipid soluble drugs with prolonged half-life and increased oral bioavailability were proposed to explain why elderly people have different pharmacokinetics compared to younger adults and these causes could potentially contribute to the differences in plasma concentration in Cats D and E [53,54]. However, an increase in volume of distribution and prolonged half-life were not observed in the older cats (Cats A, B, C and G). In humans, while mefloquine blood concentrations during pregnancy are lower than those in non-pregnant adults, no age-related differences in mefloquine plasma concentrations were found in pharmacokinetic profiles [55,56]. Interestingly, the maximum blood concentrations are 2–3 times higher in Asian adults compared to non-Asian volunteers, the reason for this ethnic difference is unclear [30,57]. It has been suggested that the smaller volume of distribution secondary to a lower body fat content or differences in enterohepatic circulation of the drug in Asian volunteers could have contributed to the higher plasma concentrations [58]. In our study, the differences in plasma concentrations between gender, neutering status and body weight could not be identified. Cat E also had normal biochemical analytes pre-treatment, at 168 and 336 h. Liver dysfunction causing altered metabolism of the drug is unlikely; however, it cannot be completely excluded without the pre- and post-prandial bile acid level to assess liver function. In people, mefloquine is metabolised by cytochrome P450 3A (CYP 3A) in the liver [59]. In cats, CYP 3A activities have been found to be lower in female cats compared to male cats [60]. However, the odd plasma concentration curve of Cat E cannot be explained. Another explanation could be that Cat E might have vomited after the first mefloquine administration without being observed. 

A difference in pharmacokinetic profile is observed in healthy vs humans infected with malaria. In humans, mefloquine plasma concentrations are 2–3 times higher in uncomplicated falciparum malaria when compared to healthy volunteers. Uncomplicated falciparum malaria also has a reduced half-life [30,31,33,61]. The cause is not entirely understood. One possible cause of the reduced half-life in malaria patients is the decrease in enterohepatic circulation and greater faecal clearance. Another explanation of the variation in pharmacokinetic profile between the two groups is the difference in the plasma protein binding of mefloquine. Mefloquine extensively binds to plasma proteins, particularly acute phase proteins such as alpha-1-acid glycoprotein (AGP) [62]. The increase in AGP in malaria is thought to result in increased mefloquine plasma protein binding, thus affecting the apparent volume of distribution [61]. High levels of AGP have been demonstrated in experimentally induced FIP [63] and naturally infected FIP cats [64,65], and is commonly used as a diagnostic tool for FIP in practice [66]. Thus, it is possible that the high level of AGP and potentially other acute phase proteins in FIP infected cats increases mefloquine plasma protein binding, altering the pharmacokinetic profile in these cats. The plasma protein binding of mefloquine in clinically normal and FIP infected cats has been explored in vitro; however, the difference was equivocal due to the unknown biological variability of the assay [19]. Further studies on pharmacodynamics and pharmacokinetics of mefloquine are needed in cats with FIP. 

An increase trend in SDMA with no change in creatinine was observed in all cats (Figure 3) during this study. SDMA has been shown to be an early renal biomarker compared to creatinine [67,68,69] and increases in acute kidney injury and chronic kidney disease [70]. Increased serum SDMA concentrations were associated with reduced renal function measured by glomerular filtration rate (GFR) in cats [71]. Based on the result of our study, it is possible that mefloquine may cause reduced renal function in cats. Renal toxicity from anti-malarial drug administration is rare in people [72]. The other explanation of increased SDMA concentrations is the influence of general anaesthesia and the cumulative effect of isoflurane during the study. Serum SDMA levels measured after anaesthesia induction (17.11 g/dL) have been shown to be significantly higher than those measured before anaesthesia induction (12.39 g/dL) [73]. As the cats were anaesthetised with isoflurane during blood collections, this could potentially contribute to the increase in SDMA concentrations. The blood collection method, mefloquine dosing and recruitment of cats was outsourced to an external designated animal research facility (Invetus Pty, Ltd.,Casino, NSW, Australia) because of lack of subject availability and this study was conducted according to Invetus’ standard operating procedures. 

One of the limitations of this study was that not all cats had normal liver enzymes pre-treatment. Three cats had elevated ALT, AST and ALP levels pre-treatment. As the blood collection, pre-treatment blood tests and treatment were performed at an external facility, the investigators did not know about the elevated liver parameters prior to treatment. The investigators were also not involved in the recruitment process of these cats, and the drug histories and previous disease records of these cats were unknown. Despite this, a significant increase in ALT and ALP after mefloquine treatment did not occur. ALT and ALP remained unchanged at 336 h in one cat (Cat G). The mefloquine plasma concentration curve of Cat G did not differ substantially from the other cats (Cat A, B and D). 

An additional limitation is the small number of cats in our study. Only five cats had complete mefloquine plasma concentrations and only four cats were included in the pharmacokinetic analysis (low concentration curve of cat E was omitted). Despite the small number of cats used in the analysis, an important description of mefloquine’s drug profile in the clinically normal cat was provided. This preliminary information is crucial for any further research projects that involve mefloquine use in cats. 

## 5. Conclusions

The study provides preliminary data for the pharmacokinetic profile of mefloquine in cats and provides useful information for the planning of clinical trials of mefloquine to treat cats with feline coronavirus (including FIP) and feline calicivirus infections and possibly, if the need arises, for COVID-19 to potentially reduce viral shedding. Further studies on the therapeutic effects of mefloquine in cats with these diseases are needed to determine its therapeutic advantage.

## Figures and Tables

**Table 1:** Mefloquine plasma concentrations of individual cats. Cat C and F were excluded from pharmacokinetic (PK) analysis due to incomplete data. Cat C was removed from the study due to vomiting, while Cat F vomited after the first treatment but was successfully re-introduced into the study on treatment day 2. Cat E was excluded from PK analysis because this cat’s profile was an outlier and skewed the data.

Mefloquine Plasma Concentrations ug/mL							
H	Cat D	Cat A	Cat B	Cat G	Cat E	Cat C	Cat F
	Male Neutered	Male Neutered	Female Neutered	Female Neutered	Male Neutered	Female Neutered	Female Neutered
0	0.00	0.00	0.00	0.00	0.00	0.00	0.00
1	0.54	1.58	0.31	1.97	0.22	0.86	-
2	0.95	1.95	0.89	2.79	0.40	2.19	-
4	1.13	2.10	1.84	3.23	0.49	-	-
8	1.59	2.23	2.20	2.89	0.64	-	-
12	1.77	2.97	2.14	3.22	0.72	-	-
24	2.09	3.35	2.09	3.12	0.56	-	-
48	1.86	3.34	1.97	2.88	0.56	-	-
96	1.51	2.76	1.75	2.50	0.57	-	-
168	2.75	4.15	3.36	3.73	3.58	-	1.39
240	1.85	6.51	2.93	4.94	4.20	-	1.54
336	3.07	4.19	3.60	4.56	2.09	-	2.11

**Table 2:** Pharmacokinetics indices of Cats A, B, D and G over the first 96 h.

PK Indices	Units	Mean	SD	Median	Min	Max
ke _(48–96 h)_	1/h	0.003	0.001	0.003	0.003	0.005
t1/2	h	224.18	51.60	233.94	153.24	275.60
Tmax	h	15.00	10.52	16.00	4.00	24.00
Cmax	μg/mL	2.71	0.66	2.71	2.09	3.35
AUC _0–96 h_	μg/mL × h	228.30	62.23	228.18	166.59	290.25
AUMC _0–96 h_	μg/mL × h^2^	10737	2971.7	10576	7826.0	13968
MRT _0–96 h_	h	326.50	13.60	337.46	221.17	397.47
V/F_obs (as calculated over 0–96 h)_	L/kg	17.41	4.08	15.74	14.73	23.41
Cl/F_obs (as calculated over 0–96 h)_	L/h/kg	0.06	0.02	0.052	0.04	0.085

SD—standard deviation; ke—elimination rate constant; t1/2—elimination half-life; Tmax—time to reach peak plasma concentration; Cmax—peak plasma concentration; AUC _0–96 h_—area under the curve over 96 h; AUMC _0–96 h_—area under the moment curve over 96 h; MRT _0–96 h_—mean residence time; V/F_obs_—apparent volume of distribution; Cl/F_obs_—apparent clearance.

**Table 3:** Biochemistry results (mean and range) at 0, 168 and 336 h. Numbers outside reference intervals are emboldened. Not all biochemical analytes were provided due to the availability of different biochemical analytes at different laboratories, hence the blank cells.

Biochemical Analyte		0 h		168 h		336 h		Reference Interval (Idexx Reference Laboratory)
	Units	Mean	Range	Mean	Range	Mean	Range	
Glucose	mmol/L	-	-	5.10	3.90–6.30	4.50	3.40–5.40	3.20–7.50
SDMA	ug/dL	6.70	1.00–8.00	11.0	8.00–13.0	**13.5**	**10.0–16.0**	0.00–14.0
Creatinine	umol/L	115	90.0–140.	122	80.0–160.	120.	100.–140.	80.0–200.
Urea	mmol/L	8.00	6.80–10.2	7.70	6.90–9.10	8.08	6.90–9.20	5.00–15.0
Phosphorus	mmol/L	1.70	1.40–2.00	1.40	1.17–1.63	1.32	1.20–1.60	0.00–2.30
Calcium	mmol/L	2.40	2.30–2.50	2.40	2.40–2.60	2.30	2.20–2.40	2.10–2.80
Sodium	mmol/L	152	149–153	154	152–156	151	148–153	144–158
Potassium	mmol/L	5.10	4.50–5.20	4.50	4.10–5.20	4.40	4.10–4.70	3.70–5.40
Chloride	mmol/L	115	111–117	123	120–125	118	116–120	106–123
Bicarbonate	mmol/L	16.0	15.0–18.0	-	-	16.3 (4 cats)	15.0–18.0	12.0–24.0
Anion gap	mmol/L	25.8	25.2–27.1	-	-	20.6 (4 cats)	20.1–21.3	15.0–31.0
Total protein	g/L	**75.3**	**67.0**–**86.0**	71.0	65.2–80.7	71.3	65.0–83.7	60.0– 84.0
Albumin	g/l	31.7	29.0–36.0	28.9	27.8–30.0	30.0	28.0–32.0	25.0–38.0
Globulin	g/L	43.7	35.0–37.0	**42.3**	**35.3–52.9**	**41.2**	**33.0–55.7**	31.0–52.0
ALT	U/L	**79.2**	**43.0**–**161**	**47.3**	**26.0–116**	**58.7**	**22.0–166**	19.0–100.
AST	U/L	**47.8**	**25.0–83.0**	29.7	22.0–53.0	29.8	20.0–42.0	0.00–62.0
ALP	U/L	**39.8**	**21.0–75.0**	**37.0**	**24.0–72.0**	**41.8**	**27.0–87.0**	5.00–50.0
GGT	U/L	0.70	0.00–1.00	-	-	0.50	0.00–2.00	0.00–5.00
Total bilirubin	umol/L	3.00	3.00	2.10	0.40–2.70	2.60	2.00–3.00	0.00–7.00
Cholesterol	mmol/L	3.85	2.60–4.90	3.90	3.00–4.80	3.10	0.00–4.70	2.20–5.50
CK	U/L	**299**	**133**–**735**	178	97.0–398	148	17.0–248	64.0–400
TT4	nmol/L	32.5	21.0–39.0	32.6	28.6–36.7		32.4–35.4	10.0–60.0

SDMA—symmetric dimethylarginine; ALT—alanine transaminase; AST—aspartate transaminase; ALP—alkaline phosphatase; GGT—gamma-glutamyl transpeptidase; CK—creatine kinase; TT4—total thyroxine. Numbers outside reference intervals are bolded.
